# Successful management of an infant with hypertensive heart failure associated with Wilms’ tumor: a case report

**DOI:** 10.1186/s40981-020-00318-7

**Published:** 2020-02-13

**Authors:** Hiroko Miura, Shin Kawana, Shigekazu Sugino, Chika Kikuchi, Masanori Yamauchi

**Affiliations:** 1 Department of Anesthesia, Miyagi Children’s Hospital, 3-17, Ochiai 4, Aoba-ku, Sendai, Miyagi 989-3126 Japan; 2 Department of Anesthesiology and Perioperative Medicine, Tohoku University School of Medicine, 2-1, Seiryo-machi, Aoba-ku, Sendai, Miyagi 980-8575 Japan

**Keywords:** Wilms’ tumor, Hypertension, Heart failure, Renin-angiotensin-aldosterone system

## Abstract

**Background:**

Wilms’ tumor with hyperreninemia may result in critical cardiovascular decompensation. We report a case of severe hypertensive heart failure followed by tumor resection in a 3-month-old infant with Wilms’ tumor.

**Case presentation:**

A 3-month-old girl was admitted to the intensive care unit for Wilms’ tumor with hypertension and hypoxia. Her systolic blood pressure was 110 mmHg, and her SpO_2_ was 92%. She presented with severe hypertensive heart failure and received mechanical ventilation and antihypertensive therapy for hypertension and heart failure. An alpha 2-adrenergic receptor agonist was used for sedation as part of her antihypertensive therapy. On hospital day 16, nephrectomy with tumor resection was performed under general anesthesia. Her systolic blood pressure did not vary more than 20 mmHg during surgery due to appropriate preoperative management. Hemodynamic collapse did not occur.

**Conclusions:**

The highlight of this case report is the successful management of an infant with Wilms’ tumor, particularly with respect to preoperative hemodynamic control and sedation.

## Background

The prevalence of hypertension is over 50% in children with Wilms’ tumor [1]. This secondary hypertension results from an increased plasma concentration of renin, which is produced by areas of the kidney cortex entrapped within the tumor [2–5]. Hypertension associated with Wilms’ tumor may progress from cardiac hypertrophy to critical cardiovascular decompensation [6–8]. We report a case of severe hypertensive heart failure in a 3-month-old infant with Wilms’ tumor. She required mechanical ventilation and drug therapy for heart failure in an intensive care unit (ICU) before tumor resection surgery under general anesthesia. This case report was prepared following the CARE guideline [9].

## Case presentation

A 3-month-old girl (Asian, 6.4 kg, 61.0 cm) presented to the emergency department with pallor, anorexia, hypotonia, and tachycardia with a heart rate of 190 beats/min. She also had hypertension with a systolic blood pressure of 110 mmHg, and hypoxia with an SpO_2_ of 92%. A large mass (57 × 52 mm) was palpable at the upper left abdomen. She had a cleft palate. Immediately after obtaining a medical history and physical examination, the patient was severely hypoxic with an SpO_2_ of 70%. The patient was immediately intubated and transferred to the ICU.

**Fig. 1:**
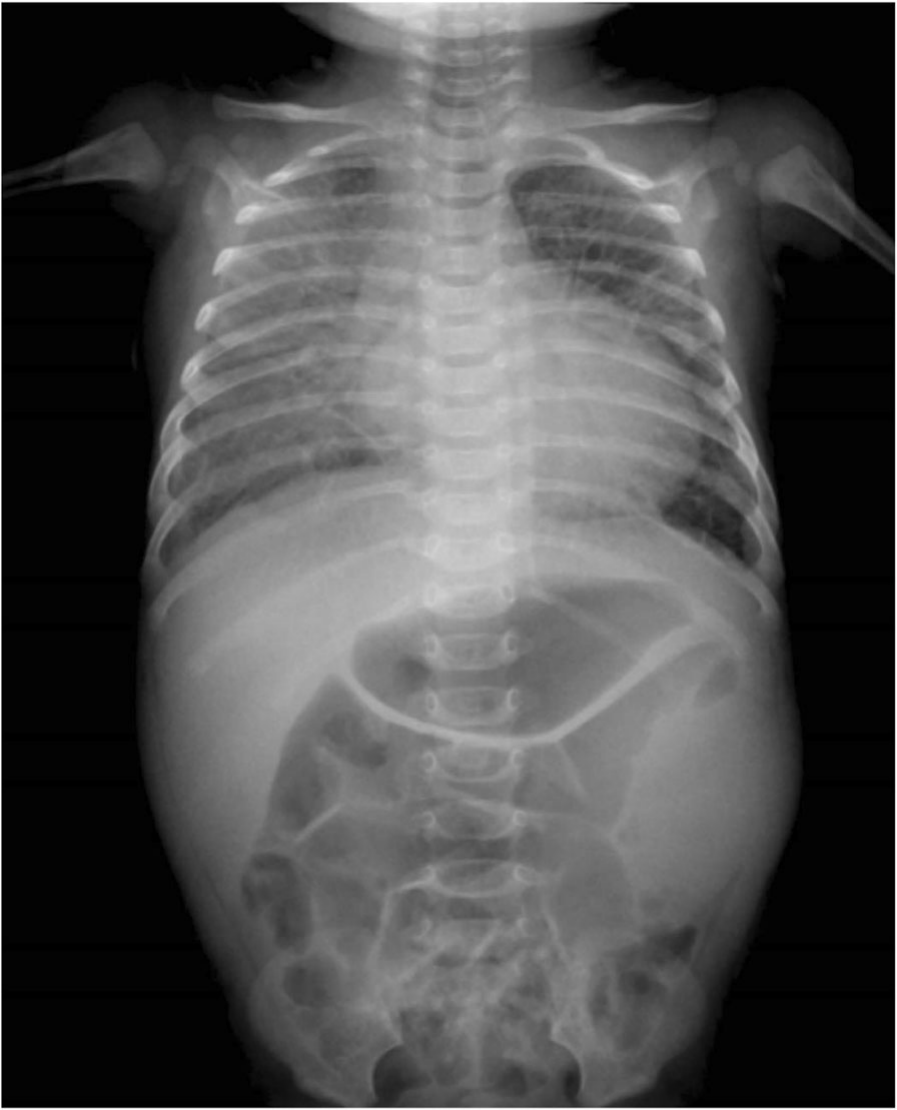
A chest X-ray showed cardiomegaly and pulmonary edema on admission to the ICU

She received mechanical ventilation immediately after admission to the ICU. A chest X-ray showed cardiomegaly with an increased cardiothoracic ratio of 54% and pulmonary edema (Fig. 1). Cardiac ultrasound showed a reduced ejection fraction of 20%. Arterial blood gases at an FiO_2_ of 40% were pH 7.53, PaO_2_ 88.4 mmHg, PaCO_2_ 35.5 mmHg, and HCO_3_^−^ 29.6 mmol/L. Blood tests showed an elevated B-type natriuretic peptide (BNP) of 3305.4 pg/mL; the concentrations of renin, angiotensin I, angiotensin II, and aldosterone were 222.6 ng/mL, 12,421 pg/mL, 388 pg/mL, and 539.7 ng/dL, respectively. Milrinone was infused at a rate of 0.5 μg/kg/min, and 30 mg of furosemide and 15 mg of potassium canrenoate were administered.

On hospital day 2, the arterial blood gas measurements at an FiO_2_ of 40% were pH 7.50, PaO_2_ 126 mmHg, PaCO_2_ 42.2 mmHg, and HCO_3_^−^ 32.2 mmol/L. The patient was successfully extubated. After 36 h, she was transferred to the floor. On hospital day 4, her systolic blood pressure increased to >140 mmHg. Twenty milligrams of nifedipine, 0.5 mg of lisinopril, and 180 μg of clonidine were administered orally. On hospital day 5, the systolic blood pressure was stabilized between 80 and 100 mmHg. The ejection fraction improved to 52%, and BNP fell to 47 pg/mL. Since the hypertensive heart failure was controlled, abdominal tumor resection by radical nephrectomy was scheduled. Milrinone was discontinued on hospital day 15. Her systolic blood pressure was 90 mmHg before surgery.

**Fig. 2:**
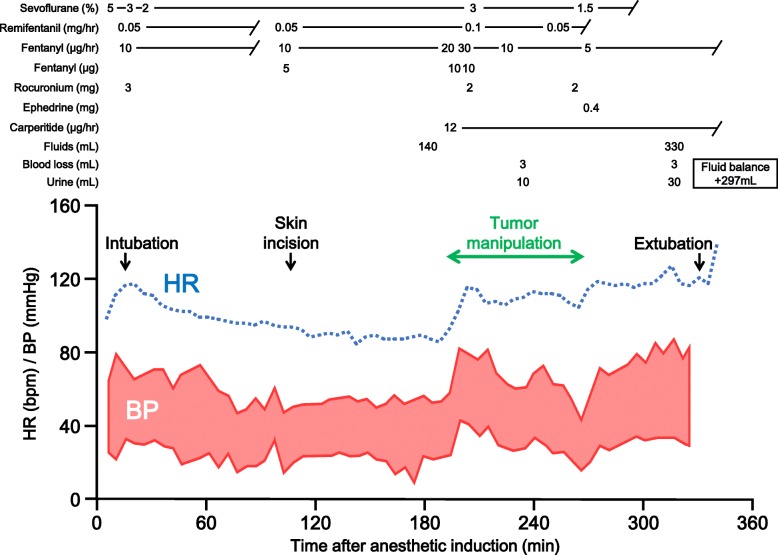
Anesthesia record of an infant who underwent Wilms’ tumor resection. HR: heart rate, BP: blood pressure

On hospital day 16, the patient was transferred to the operating room. As shown in Fig. 2, general anesthesia was induced by inhalation of 5% sevoflurane. Anesthesia was maintained with 2% sevoflurane in 40% oxygen, a total of 77 μg of fentanyl, and remifentanil infusion at a rate of 0.13 μg/kg/min. After tracheal intubation, arterial and central venous catheters were placed. Carperitide was infused at the rate of 0.03 μg/kg/min to reduce left ventricular afterload and increase renal blood flow. At the time of the skin incision, the heart rate and systolic/diastolic blood pressure were 87 beats/min and 60/31 mmHg, respectively.

The systolic blood pressure increased from 60 to 80 mmHg by manipulation of the tumor (Fig. 2). After resection, the blood pressure decreased to 50 mmHg (Fig. 2). The patient needed 0.4 mg of ephedrine to maintain her hemodynamics. The operative time was 182 min. The patient was successfully extubated after emergence from general anesthesia and then transferred to the floor. Histopathological analysis demonstrated that the resection specimen was nephroblastoma (Wilms’ tumor, stage I). Adjuvant chemotherapy was used for tumor regression. The rest of her hospital stay was uneventful, and she was discharged on hospital day 66 in stable condition.

## Discussion

Wilms’ tumor with hyperreninemia produces vasoconstriction and fluid retention that leads to sustained hypertension, and patients can develop lethal congestive heart failure [10]. In our infant, milrinone and diuretics were used for congestive heart failure in the ICU. However, her hypertension could not be controlled, and we needed a calcium-channel blocker and angiotensin-converting enzyme inhibitor. For many years, several drugs have been used to treat hypertension associated with Wilms’ tumor [10]. Previous reports indicated that an angiotensin-converting enzyme inhibitor (e.g., captopril), angiotensin II analog (e.g., saralasin), or angiotensin II receptor antagonist (e.g., losartan) were administered to control severe hypertension in patients with Wilms’ tumor [8, 11, 12]; however, these drugs had limited ability to control hypertension. Since we needed to optimize antihypertensive therapy in our patient, we used clonidine, an α2-adrenergic receptor agonist, to achieve light sedation. Surprisingly, this sedation decreased the blood pressure of the infant. We speculate that any sedative may be useful for the management of hemodynamic stability in combination with antihypertensive drugs, such as angiotensin-converting enzyme inhibitors. Our preoperative medical management to achieve hemodynamic stability contributed to stable anesthetic induction and maintenance of the patient.

In our anesthetic management, small hemodynamic changes occurred before and after tumor resection. The blood pressure slightly increased despite gentle handling of the tumor and decreased after resection, regardless of the blood loss and the depth of anesthesia. These changes are similar to those that occur with surgical resection of pheochromocytoma. Charlton et al. demonstrated that infusions of phentolamine, phenoxybenzamine, and sodium nitroprusside were useful for intraoperative control of blood pressure associated with Wilms’ tumor [6]. Alpha-adrenergic blockade is actually used in surgery for pheochromocytoma resection [13]. We could have used these drugs for the management of blood pressure in our surgery. With regard to the small decrease in blood pressure, intermittent mechanical compression of the inferior vena cava by the surgeon may induce hypotension. Another possible explanation is that hypovolemia from hemorrhage and downregulation of angiotensin receptors contribute to hypotension. This possible mechanism is consistent with the observation that hemorrhage and downregulation of α- and β-adrenergic receptors induce hypotension after pheochromocytoma resection [13]. However, we did not measure the concentrations of renin, angiotensin I, angiotensin II, and aldosterone during surgery before or after tumor resection. The underlying mechanism of hypotension in our infant remains unknown.

In neonates and infants, the myocardium has less contractile force than in adults because of the less compliant ventricles [14]. This developmental immaturity of the heart accounts for their sensitivity to volume loading and poor tolerance of increased afterload. In our patient, the myocardium was continually remodeled by hypertension with hyperreninemia, and there was a tendency toward heart failure in utero and throughout her life. As mentioned above, the preoperative hemodynamic control (i.e., decreased preload and afterload) contributed to the improvement in cardiac function. We speculate that she was hypovolemic just before surgery because of the medications given to treat heart failure. However, we did not accurately evaluate intravascular volume before and during the surgery, in part because the clinical assessment of hemodynamic status based on routine monitoring or goal-directed fluid therapy by measuring aortic blood flow is limited in pediatric patients [15]. In this case, it might have been possible to use transesophageal echocardiography or non-invasive cardiac output monitoring by electrical velocimetry for precise hemodynamic control [16]. However, their usefulness in infants is still controversial.

In this case, the stage of the Wilms’ tumor was low grade, and the infant has a good prognosis. The National Wilms’ Tumor Study Group in the USA recommends five classifications (stage I–V) for Wilms’ tumor [10]. Importantly, when the stage of the tumor was higher than stage III, intravascular extension into the inferior vena cava with thrombosis frequently occurred [17]. Anesthetic management in patients undergoing Wilms’ tumor resection is of major concern. Anesthesiologists should consider the presence of pulmonary embolism, obstruction of the vena cava, or obstruction of the tricuspid valve [18]. Transesophageal echocardiography may be useful to evaluate intravascular extension during the perioperative period if the stage of the tumor is advanced.

This case report indicates that optimized preoperative hemodynamic control and sedation produce stable anesthetic management during surgery in the pediatric patient with severe heart failure associated with Wilms’ tumor.

## Data Availability

Not applicable

## Abbreviations

BNP: B-type natriuretic peptide
ICU: Intensive care unit
